# Use of traditional herbal medicine as an alternative in dental treatment in Mexican dentistry: a review

**DOI:** 10.1080/13880209.2017.1347188

**Published:** 2017-07-25

**Authors:** Cindy Cruz Martínez, Martha Diaz Gómez, Myung Sook Oh

**Affiliations:** a Department of Oriental Pharmaceutical Science, College of Pharmacy, Kyung Hee University, Seoul, Republic of Korea;; b Deparment of History, College of Dentistry, National Autonomous University of Mexico (UNAM), Coyoacan, Mexico;; c Department of Life and Nanopharmaceutical Sciences, Graduate School, Kyung Hee University, Seoul, Republic of Korea

**Keywords:** Mexican herbs, oral disease, dental herb therapy

## Abstract

**Context:** Herbal therapies are used worldwide to treat health conditions. In Mexico, generations have used them to treat gingivitis, periodontitis, mouth infections, and discoloured teeth. However, few studies have collected scientific evidence on their effects.

**Objective:** This study aimed at searching and compiling scientific evidence of alternative oral and dental treatments using medicinal herbs from Mexico.

**Methods:** We collected various Mexican medicinal plants used in the dental treatment from the database of the Institute of Biology at the National Autonomous University of Mexico. To correlate with existing scientific evidence, we used the PubMed database with the key term ‘(scientific name) and (oral or dental)’.

**Results:** Mexico has various medical herbs with antibacterial and antimicrobial properties, according to ancestral medicinal books and healers. Despite a paucity of experimental research demonstrating the antibacterial, antimicrobial, and antiplaque effects of these Mexican plants, they could still be useful as an alternative treatment of several periodontal diseases or as anticariogenic agents. However, the number of studies supporting their uses and effects remains insufficient.

**Discussion and conclusion:** It is important for the health of consumers to scientifically demonstrate the real effects of natural medicine, as well as clarify and establish their possible therapeutic applications. Through this bibliographical revision, we found papers that testify or refute their ancestral uses, and conclude that the use of plants to treat oral conditions or to add to the dental pharmacological arsenal should be based on experimental studies verifying their suitability for dental treatments.

## Introduction

Humans have sought cures for diseases in nature since ancient times; even recently, the use of herbal medicines in dietary supplements, energy drinks, multivitamins, massage, and weight loss products has gained popularity (Petrovska 2012). These uses have broadened the field of herbal medicine and also increased its credibility.

The field of dentistry also has begun to exploit herbal properties for the purpose of relieving tooth pain, gum inflammation, and canker sores (Kumar et al. 2013). However, it is of utmost importance to understand the interactions of plant extracts with the body and other medications, as many of these extracts have anti-inflammatory effects and prevent bleeding, which is important in dental treatment (Taheri et al. 2011). Antiseptics, antibacterial, antimicrobial, antifungal, antioxidant, antiviral, and analgesic agents derived from plants are of widespread interest in dentistry (Sinha and Sinha 2014). For example, in recent years, in the field of periodontics and endodontics, several plant extracts such as a propolis, noni fruit, burdock root, and neem leaf have been used as intra-canal medications with excellent results, opening up a novel function for herbal agents in global dental therapy (Pujar and Makandar 2011; Shah et al. 2015).

In Mexico, the Aztec and Mayan cultures developed many uses for medicinal plants (Galarza 1981); this development ceased after the conquest, when the Spaniards controlled and evangelized the Aztecs (Cortez et al. 2004). The Spaniards introduced new products from the Old World to Mexico and, combined with native methods, thus enriched the natural medicine arsenal (Garcia 1991). Historical knowledge is essential because, without it, we would lack clarity and our medical practices would lack coherence (Estrada 1996). The effectiveness and possible application of numerous Mexican medicinal plants has not yet been studied with respect to dentistry. Dental services even in the urban and in the rural areas of Mexico are expensive, and it is difficult for people to access the appropriate drugs (Medina-Solis et al. 2006; Maupome et al. 2013). For these reasons, herbal remedies in Mexico are commonly used despite the lack of scientific support for their use, dosage, and effects (Andrade-Cetto 2009). In fact, people use them without caution because they believe such alternative treatments have no risks or no possibility of allergic reactions or other adverse effects as they come from natural sources. Therefore, it is important to study, analyse, and test the efficacy of traditional medicinal plants to establish and promote their use as alternative treatments or as potential sources for obtaining or developing new drugs.

This study describes and clarifies the types of alternative oral and dental treatments based on herbal therapies that are commonly used in Mexico. We also reviewed the limited experimental evidence regarding herbal therapy to support the use of traditional Mexican medicine as a possible aid in the treatment of dental and oral pathologies, as well as a potential source for the development of drugs.

## Literature search

We collected the various Mexican medicinal plants used in dental treatment from the database of the Institute of Biology at the National Autonomous University of Mexico (Digital Library of Traditional Mexican Medicine; DLTMM). We searched the electronic literature in the PubMed database with the keyword ‘(scientific name) and (oral or dental)’ to correlate with existing scientific evidence on the Mexican plants.

## Uses of herbal medicines in ancient Mexican cultures

The rise of Mexican medicine occurred during the Aztec and Maya empires and all or almost all of information on these ancestral medicinal skills was collected in codices by religious orders, such as the Franciscans (Galarza 1981; Garcia 1991). Aztec medicine had a magical-religious approach to healing or the treatment of disease (Estrada 1985). Using the same approach, the medicinal skills of Mayans included methods to heal wounds and counter rattlesnake venom, massage techniques to restore dislocations or banish inflammation, hot baths involving herbal steam cooking, and the use of pricks from porcupine spines to treat neuralgia, similar to the principle of Chinese acupuncture (Berdaguer 1991; Cañigera et al. 2003; Santana et al. 2015). With regard to oral or dental treatments, the Mayans used quartz powder as an abrasive to clean out carious cavities before sealing them with a powder mixture that had a high resistance to mastication (De la Cruz 1975). For the treatment of the dental pain, they used the root of Chicalote (*Argemone Mexicana* L. [Papaveraceae]) as a reliable anaesthetic (Galarza 1981; Estrada 1996; Cortez et al. 2004).

The Florentine Codex, which was written in Náhuatl, the native language, and translated into Spanish by Fray Bernardino de Sahagún in 1557, describes the names and uses of many medicinal plants and animal materials (Galarza 1981; Terraciano 2010). The *Libelus de medicinabilus indorum herbis* was written by Martín de la Cruz, an indigenous Mexican doctor, and translated by Juan Badiano from Náhuatl into Latin. It contains descriptions of herbs’ effects and their applications along with colour illustrations, covering all diseases of the human body by beginning with the head and ending with the signs of death. It includes a section on oral health and dental conditions, and ultimately paints a holistic view of stomatology (De la Cruz 1975; Garcia 1991; Estrada 1996; Salas and Rivas 2001). In 1712, the *Anthology Medicinal* also described many Mexican herbal dental treatments (Rojas 2009).

Despite the fact that Mexico is rich in medicinal plants, this area of medicine has not been completely developed, or at least, is not a priority in Mexican medicine (Lautie et al. 2008). Herbal culture is transmitted orally from generation to generation (De la Rosa 1980). Herbal products are preferred over prescription medications for treating certain illnesses because of their lower cost or because people may believe the herbs to be less toxic, given that they are natural (Rivera et al. 2005a; Brindis et al. 2013). Generally, people visit the doctor only if they do not respond to home remedies (Waldstein 2008). In rural communities, traditional medicine is the best choice for the people, even if the community has medical services (Arrieta-Baez et al. 2012). A study of the use of complementary and alternative medicine among Hispanics found that the most commonly reported alternative therapies were herbs, prayer, and dietary supplements (Mikhail et al. 2004). Mexican street markets offer plants that are used as analgesics, anti-inflammatory treatments, and antiseptics, as well as treatments for pathologies as varied as scorpion stings and cancer (Josabad Alonso-Castro et al. 2012). Medicinal plants are used for a wide variety of purposes and are traded both nationally and internationally (Moreno et al. 2006).

## Traditional uses of Mexican herbs in dentistry

In Mexico, the most common oral diseases are caries and periodontal disease. However, dental services in rural areas are very expensive and do not represent a primary health concern for rural people, who prefer to use alternative medicine for this common but simple oral disease. Approximately 59.6% of people in Mexico have signs of periodontal disease and the prevalence of caries in the population over age 40 is close to 97% (Cruz and Picazzo 2017). The method of preparation of medicinal plants varies depending on the kind of plant, as well as the portions used (stems, leaves, and roots), route of administration (local, topical, and rinse), and time of ingestion. In some areas, people who have dental pain prepare fillings from a plant or chew the bark of multiple trees to treat inflammation, as well as use plant extracts as mouthwashes or teas.

The use of medicinal plants can be an advantage in dental practice, for example eugenol is a part of our therapeutic arsenal (Rojas 2009; Da Silva et al. 2012). Some herbal products have recently undergone a thorough investigation with regards to their potential for preventing oral diseases, such as dental caries (Moreno et al. 2006). Although many years had elapsed without research on medicinal plants, this trend reversed when the National Medical Institute was established in 1888, creating new possibilities for herbal remedies (Rojas 2009; De Micheli-Serra and Izaguirre-Avila 2014). Because plants are often the sources for novel drugs, their screening should be a priority in drug development (Lautie et al. 2008).

Medicinal plants are an important element of indigenous medical system in Mexico (Heinrich 2000). However, interest in their effects and subsequent demonstrative studies are lacking. Table 1 presents a summary of the plants in DLTMM that are either used in Mexico or are of Mexican origin and used elsewhere for oral disease.

**Table 1.** Mexican plants used in the treatment of the oral disease from the Digital Library of Traditional Mexican Medicine.

Scientific name (Family name)	Common name	Used part	Indications
*Acacia cornigera* (L.) Willd (Leguminosae)	Cornezuelo	Leaf	Inflammation of gums
*Acacia farnesiana* (L.) Willd. (Leguminosae)	Huizache	Stem	Cold sore and toothache
*Amphipterygium adstringens* Schiede ex Schlech. (Anacardiaceae)	Cuachalalate	Latex	Peridontitis
*Asclepias curassavica* L. (Asclepiadaceae)	Quiebra muelas	Latex	Caries and toothache
*Bidens odorata* Cav. (Compositae)	Aceitilla	Leaf	Canker sores
*Byrsonima crassifolia* (L.) Kunth (Malpighiaceae)	Nanche	Leaf and flower	Toothache
*Caesalpinia pulcherrima* (L.) Swartz (Leguminosae)	Tabachin	Fruit and root	Canker sores
*Capsicum frutescens* L. (Solanaceae)	Chile de arbol	Leaf	Toothache
*Carica papaya* L. (Caricaceae)	Papaya	Leaf and fruit	Canker sores
*Chenopodium graveolens* (Willd.) Weber (Chenopodiaceae)	Epazote	Leaf	Toothache
*Chiranthodendron pentadactylon* Lam. (Sterculiaceae)	Flor de manita	Flower	Toothache
*Dorstenia contrajerva* L. (Moraceae)	Contrayerba	Root	Caries, toothache, and tooth abscess
*Heterotheca inuloides* Cass. (Compositae)	Arnica	Flower	Canker sores
*Heliopsis longipes* (A. Gray) S.F. Blake. (Asteraceae)	Chilcuague	Root	Toothache
*Jatropha gaumeri* Greenm. (Euphorbiaceae)	Pomolche	Latex or leaf	Canker sores, oral candidiasis, and tooth abscess
*Lobelia laxiflora* Kunth. (Campanulaceae)	Aretillo or zarcillo	All plant	Canker sores and toothache
*Opuntia ficus-indica* (L.) Miller (Cactaceae)	Nopal	Fruit and flower	Oral ulcer and tooth abscess
*Persea americana* Miller. (Lauraceae)	Aguacate	Fruit	Canker sores, gingivitis, periodontal disease, and toothache
*Sida rhombifolia* L. (Malvaceae)	Escobilla or malvilla	Stem and leaf	Gingivitis and toothache
*Theobroma cacao* L. (Sterculiaceae)	Cacao	Bean	Oral ulcer and toothache

Dentistry is seeking novel and effective alternative healing techniques. One possible approach is to review historical data and evaluate how people of the past cured oral disease. Through such review and analysis, new horizons in dentistry and other fields of medicine may be reached.

## Experimental evidence related to the use of Mexican herbs in dentistry

Although Mexico has a great diversity of medicinal plants, research to confirm or refute their popular uses has been very limited. However, due to the popularity of these plants in different countries, we have developed great interest in learning more about Mexican medicine. Table 2 presents a summary of the plants that are used in Mexico for oral disease with experimental evidence.

**Table 2.** Mexican plants used in the treatment of the oral disease according to experimental evidence.

Scientific name (Family name)	Subjects	Outcomes	Reference
*Aloe vera* (L.) Burm.f. (Asphodelaceae)	120 volunteers with gingivitis aged 18–25 years old	Inhibition of gingivitis and plaque accumulation after oral rinse	Chandrahas et al. (2012)
	45 patients with plaque-induced gingivitis aged 18–65 years old	Reduction of gingival inflammation	Ajmera et al. (2013)
	345 healthy subjects	Reduction of gingival bleeding and plaque indices	Karim et al. (2014)
	76 intubated patients in intensive care unit aged 18-64 years old	Reduction of gingival index compared with chlorhexidine	Rezaei et al. (2016)
	390 healthy subjects	Reduction of gingival index compared with chlorhexidine	Vangipuram et al. (2016)
*Capsicum frutescens* L. (Solanaceae)	Human buccal mucosa fibroblast cell line	Suppression of cell growth and total cell death	Van Wyk et al. (1995)
*Chenopodium ambrosioides* L. (Dysphaniaceae)	Minimum bactericidal concentration determination in culture plates	Ineffective antibacterial activity against *S. mutans*	Vieira et al. (2014)
*Opuntia ficus-indica* (L.) Miller (Cactaceae)	Burning mouth syndrome patients	Amelioration of hyposalivation and mouth pain	Castillo and Aldape (2006)
*Persea americana* Miller. (Lauraceae)	Human periodontal ligament and human alveolar bone cell line	Preventive action on the deleterious effects exerted by interlukin-1beta in periodontal diseases	Andriamanalijaona et al. (2006)
	Minimum bactericidal concentration determination in culture plates	High antibacterial activity against *S. mutans* or *Porphyromonas gingivalis*	Rosas-Pinon et al. (2012)
*Polygonum aviculare* L. (Polygonaceae)	60 volunteers with gingivitis aged 18–25 years old	Inhibition of gingivitis after oral rinse	Gonzalez Begne et al. (2001)
*Punica granatum* L. (Punicaceae)	23 volunteers with gingivitis and dental plaque aged 22–28 years old	No significant activities between control and experimental groups for the visible plaque index and gingival bleeding index	Salgado et al. (2006)
*Theobroma cacao* L. (Sterculiaceae)	Caries rats induced by *S. mutans*	Reduction of caries development and dental plaque accumulation	Ooshima et al. (2000)
	Broth medium with *S. mutans*	Reduction of the growth rate of oral streptococci by decrease of acid production	
	28 volunteers with plaque depositions aged 19–29 years old	Antibacterial activity against *S. mutans*	Matsumoto et al. (2004)
	Perpendicular steel wire with artificial dental plaque	Antiplaque formation	
	The selected children with scaling of the teeth	Reduction of colonization by *S. mutans* and plaque deposition	Srikanth et al. (2008)
	50 children of both sexes aged 6–10 years old	Antimicrobial activity similar to chlorhexidine	Venkatesh Babu et al. (2011)
*Uncaria tomentosa* (Willd. ex Schult.) DC (Rubiaceae)	Minimum bactericidal concentration determination in culture plates	Higher antimicrobial activity on *Enterobacteriaceae*, *S. mutans*, and *S. aureus* isolates	Ccahuana-Vasquez et al. (2007)

Mexican *Sanguinaria* (*Polygonum aviculare* L. [Polygonaceae]), which was shown to be an anti-inflammatory, astringent, and diuretic plant, is commonly used in the treatment of gingivitis to decrease the inflammatory process (Gonzalez Begne et al. 2001). A clinical study in students between the ages of 18–25 years who used the Mexican *Sanguinaria* extract as oral rinse for 14 days found that the extract significantly decreased gingivitis from day 0 to 14 (*p* ≤ 0.05) (Gonzalez et al. 1999). A recent study demonstrated the wound healing effects of quercitrin hydrate, caffeic acid, and rutin as its active compounds (Seo et al. 2016). In 2000, the use of a paste manufactured from *Uncaria tomentosa* Willd. ex Schult. [Rubiaceae]) was compared with that of zinc oxide and eugenol for direct pulp capping (Lahoud et al. 2000). The results showed that the *U. tomentosa* paste was more efficacious, as it not only decreased pulp inflammation more effectively, but also promoted better dental reformation and was more effective against microorganisms that usually inhabit the human oral cavity; *U. tomentosa* inhibited 8% of *Enterobacteriaceae* isolates, 52% of *Streptococcus mutans*, and 96% of *Staphylococcus aureus* (Ccahuana-Vasquez et al. 2007). However, the tested concentrations did not have an inhibitory effect on *pseudomonas aeruginosa* and *Candida albicans* (Valerio and Gonzales 2005; Ccahuana-Vasquez et al. 2007).

In other studies, the effect of an Aloe (*Aloe vera* (L.) Burm.f. [Asphodelaceae]) mouthwash was investigated, as the plant has anti-inflammatory and antibacterial activities. These activities may be derived from those of aloin and emodin as active components (Surjushe et al. 2008). The antimicrobial susceptibility test showed that both the gel and the leaf inhibited the growth of *S. aureus* at 18.0 and 4.0 mm, respectively. Only the gel inhibited the growth of *Trichophyton mentagraphytes* (20.0 mm), while the leaf possesses inhibitory effects on both *P. aeruginosa* and *C. albicans* (Agarry et al. 2005). It proved its effectiveness in the treatment of gingival inflammation and led to reduced plaque (Chandrahas et al. 2012; Ajmera et al. 2013; Karim et al. 2014; Rezaei et al. 2016; Vangipuram et al. 2016).

In addition, researchers used a rat model to show that Wildemalva (*Pelargonium zonale* (L.) L'Hér. ex Aiton [Geraniaceae]), a plant known as ‘marriage or boyfriend’ (Price and Palmer 1993), has local haemostatic action to apply dental surgery, with the bleeding time 50% shorter in the leaf juice treatment group (18.10 ± 2.03 min) and 80% shorter in the crushed-leaf group (7.10 ± 0.88 min) than in the control group (37.6 ± 3.04 min) (Paez and Hernandez 2003). Meanwhile, Salgado et al. (2006) demonstrated that the antibacterial and anti-inflammatory effects of the grains and flowers of Granada (*Punica granatum* L. [Punicaceae]) were not efficient in gingivitis. The results did not show a statistically significant difference between the control and experimental groups for both visible plaque index and gingival bleeding index.

In the states of Hidalgo, Puebla, and Tlaxcala in Mexico, the ‘evergreen’ plant (*Bryophyllum pinnatum* (Lam.) Kurz. [Crassulaceae]), which has green leaves throughout the year, is used for toothache, tooth whitening, and the treatment of periodontitis (Kamboj and Saluja 2009). In a study in rats, the inhibition of the carrageenan oedema by evergreen extract at a dose of 100 mg/kg was 80%, which was improved by increasing the dose to 200 mg/kg (Dominguez and Bacallao 2002). The nopal cactus (*Opuntia ficus-indica* (L.) Miller [Cactaceae]) is also listed as one of the major components of Mexican herbology. This grows extensively throughout Mexico and is especially abundant in the arid and semi-arid regions of central Mexico (Chávez-Moreno et al. 2009). It is used for both its nutritive and hypoglycaemic properties. Several bioactive compounds such as indicaxanthin and betanin may contribute to various biological activities due to their potent anti-oxidant and anti-inflammatory actions (El-Mostafa et al. 2014). In a study in rats, it reduced postprandial blood glucose by 46.0% and 23.6%, respectively (*p* < 0.05), in comparison to the control (Nunez-Lopez et al. 2013). Its anti-inflammatory effect is used in dentistry for gingivitis, ulcers, and periodontitis (Allegra et al. 2014). In a clinical study, sialagogues therapeutics with the infusion of nopal cactus in Mexican patients was successful in combating hyposalivation and mouth pain due to viral aetiologies (Castillo and Aldape 2006). In the state of Morelos, the abrojo rojo (*Tribulus terrestris* L. [Zygophyllaceae]), a plant with plate-like flowers with five yellow petals and spiny fruits, is used three times per day as an infusion rinse to combat gingivitis (Gauthaman et al. 2002). Arnica (*Heterotheca inuloides* Cass. [Compositae]), a plant native to the hot and temperate regions of central Mexico, is commonly used as an anti-inflammatory, analgesic, and healing agent, for the treatment of contusions, skin wounds, and bruises (Martinez 1984, 1992). In cases of gingivitis, it is used as an infusion three times per day; the results have shown 96.6% effectiveness of an arnica ethanol extract, in comparison with the 66.7% effectiveness of piroxicam (Beauballet et al. 2002). In San Luis Potosi, Morelos, Puebla, and Durango, is used as an anti-inflammatory treatment, for gastrointestinal disorders (e.g., diarrhoea), oral pathologies (e.g., sore throat, sore gums), and inflammations of the breast (e.g., sore nipples), as well as for urinary tract disorders and painful rectal conditions (e.g., haemorrhoids) (Sanchez-Miranda et al. 2013). The root of the plant is also used for the treatment of stomach and bowel cancer and inflammatory conditions (Achenbach et al. 1987). The active compound of this plant is kramecyne, a potent inhibitor of iNOS, COX-2, NO, TNF-α, and IL-6 production in LPS-macrophages (Martinez 1992). In Oaxaca, Tabasco, and Aguascalientes, oak bark (*Quercus robur* L. [Fagaceae]) is used as an anti-inflammatory gingival treatment, powerful astringent for throat and mouth infections, treatment for bleeding gums, and cure for acute diarrhoea (Ernst & Lehner 2003). Many people in Mexico also use cuachalalate (*Amphipterygium adstringens* Schltdl. [Anacardiaceae]) to harden their gums, but it should be noted that excessive doses of this substance can be highly toxic (Waizel and Martinez 2011). Care is needed because it can irritate mucous membranes, however, experimental findings in rats suggest that cuachalalate methanol extract at doses lower than 100 mg/kg protects the gastric mucosa from the damage induced by diclofenac sodium without altering either the anti-inflammatory activity or the pharmacokinetics of diclofenac sodium in comparison to omeprazole, the positive control, with a strong laxative effect (Navarrete et al. 2005). In Yucatán, the papauce or anona blanca (*Annona diversifolia* Saff. [Annonaceae]) is used as food, but its leaves are employed as an anticonvulsant, analgesic, and anti-inflammatory agent (Estrada 1994). Its ethanol extract caused a 25% recovery of limb function in rats and produced a similar anti-nociceptive response (ED_50_ = 15.35 mg/kg) to that of the reference drug tramadol (ED_50_ = 12.42 mg/kg) (Carballo et al. 2010). *Castilleja tenuiflora* Benth. (Orobanchaceae) is a plant used not only for snakebites or cough, but also in the treatment of inflamed ovaries. *C. tenuiflora* was tested in a topical model of inflammation (2-O-tetradecanoylphorbol 13-acetate-induced ear oedema in mice) and found to produce a significant 20% inhibition. In contrast, indomethacin, the positive control, showed 40% inhibition (Carrillo-Ocampo et al. 2013).

Van Wyk et al. (1995) conducted a study on the effects of *Capsicum frutescens* L. (Solanaceae) on the growth of oral fibroblasts ultimately expanding the use of traditional medicine in periodontology, a field in which the use of natural agents has been very limited thus far. It is traditionally used in the treatment of toothache, gum inflammation, and dental infections by ancient Mexicans. These dental effects seem to be based on a recent research showing the antibacterial and antioxidant effects of various volatile compounds such as hexadecanoic acid (Gurnani et al. 2016). The avocado (*Persea americana* Miller. [Lauraceae]) is one of the most widely recognized Mexican medicinal plants. Rosas-Piñon et al. (2012) demonstrated the ability of avocado to inhibit the growth of the principal pathogens of periodontal disease. It also exerted inhibitory effects on the increased interlukin-1β in periodontal ligaments (Andriamanalijaona et al. 2006). Taken together, avocado could have a potential role in the prevention of oral diseases. In contrast, Vieira et al. (2014) exposed *Chenopodium ambrosioides* L. (Dysphaniaceae) as an ineffective antimicrobial agent against *S. mutans*, one of the main pathogens of the mouth. Thus, their use in the treatment of toothache is unsubstantiated. This finding illustrates that even information or ‘knowledge’ that has been transmitted generationally, needs to be evaluated and confirmed.

Even more surprising are the investigations carried out by different authors in different countries on the anti-microbial effect of cacao bean (*Theobroma cacao* L. [Sterculiaceae]), the plant from which chocolate is derived. It has been found to be a potential substitute for chlorhexidine as a mouth rinse, with powerful anticariogenic, antibacterial, and antiplaque activities (Ooshima et al. 2000; Matsumoto et al. 2004; Srikanth et al. 2008; Venkatesh Babu et al. 2011). It would be especially useful in paediatric dentistry because it would be acceptable to children without hypersensitivity and coloration of the teeth and tongue that chlorhexidine can have on children (Al-Tannir and Goodman 1994). Moreover, it has been shown that its bioactive compounds such as catechins and theobromine possess strong anti-oxidant activity (Lee et al. 2003; Ramiro-Puig and Castell 2009). Thus, *T. cacao* is a natural source of an agent with anticariogenic and potent antimicrobial activity that has potential in the field of dentistry.

## Discussion

It is well known that Mexico has a great diversity of medicinal plants (Taddei-Bringas et al. 1999). However, their uses are generally restricted to the treatment of simple diseases. In addition, only a few papers with appropriate experimental methods have been conducted on their effects. Although they lack supporting research, Mexican herbal therapies are effective; unfortunately, they do not receive validation from the medical sector, because of little or no interest. Some even believe that herbal medicine denigrates their profession.

Herbal therapy can offer many possible advantages. Some plants have been shown to be more effective than drugs at repairing the overall body due to the synergy of their active ingredients to have preventive effects, stimulate the regulatory action of the defensive functions of the body, and prepare for possible activity against external agents (Arteche 1992; Villar 2001). Side effects are often minor and therapeutic effects are more long lasting because of better tolerance and versatility (Comerford 1996). Unlike drugs that are prescribed for a specific condition, the herbal therapy may act on different targets simultaneously or acts a co-treatment with conventional medications (Cecchini 1978). The latter of course must be done carefully when combining agents without a medical indication (Fores 1997).

Herbal medicines do have some disadvantages. Depending on the type of plant, the component used, or the dose, they can be toxic. Some plants can cause abortions, interact with drugs used during surgery to prolong anaesthesia time, change vital signs, and increase postsurgical bleeding (Rivera et al. 2005a, 2005b; Albuquerque et al. 2011). Easy access to this type of medicine in Mexico is also a huge disadvantage because patients can consume medicinal plants without medical indication or supervision by an herbal therapeutic expert, leading to undesired medical interactions.

**Figure 1.**
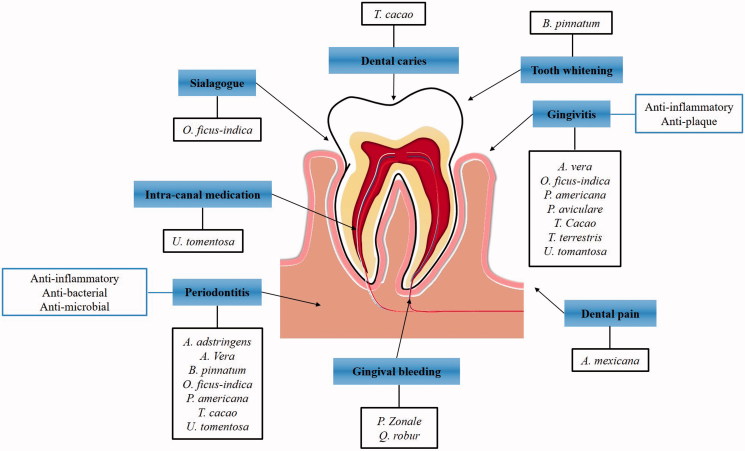
Summary of the plants that are traditionally used in Mexico or are of Mexican origin to treatment of diverse oral disease. The anti-inflammatory, anti-microbial and anti-bacterial effects of the plants are used to treatment of gingivitis, periodontitis and intra-canal medication. The anticariogenic, sialagogue and tooth whitening effect are not demonstrated yet, however, the Mexicans still used for dental treatment.

Recently, some doctors and researchers have developed an interest in discovering or confirming the therapeutic effects of Mexican medicine. For example, Arrieta-Baez et al. (2012) tested the effects of traditional Mexican medicine on gastrointestinal disorders, a major disease category in Mexico, with good results for the treatment of salmonellosis. With regards to dentistry, the use of medicinal plants as anti-inflammatory, antiseptic, or antibacterial agents has led to the development of new toothpastes and new therapeutic agents (Figure 1). Further studies are needed to support and continue this pioneering work, as it is vital for the effectiveness of these plants to be confirmed by research.

## Conclusions and perspectives

It is essential to adopt a scientific attitude toward herbal medicine: critical and skeptical, but open to new knowledge. Further research should be conducted to evaluate their effectiveness as possible pharmaceutical sources and/or support their use as treatments. At the same time, care must be taken when promoting herbal medicines because, along with their therapeutic potential, there is a risk for misuse or adulteration. Above all, it is important that effects of herbal medicine can be maximized on the basis of precise plant origin and quality control. To prevent the misuse of Mexican herbal medicine, further studies are needed to establish these conditions by each herb.

Herbal medicine is not a fad; rather, it reflects a wide and varied range of therapeutic resources, including homeopathy, acupuncture, and various forms of psychotherapy, as well as therapeutic agents derived from plants. Plants have been proposed as an alternative treatment for buco-dental diseases, a domain in which long-term reliability is an important aspect of treatment. New medical professionals must be able to assimilate popular knowledge, update it, and place it in the arsenal of modern medicine for the general benefit of society.
